# Enzymatic Degradation of Aromatic and Aliphatic Polyesters by *P. pastoris* Expressed Cutinase 1 from *Thermobifida cellulosilytica*

**DOI:** 10.3389/fmicb.2017.00938

**Published:** 2017-05-24

**Authors:** Caroline Gamerith, Marco Vastano, Sahar M. Ghorbanpour, Sabine Zitzenbacher, Doris Ribitsch, Michael T. Zumstein, Michael Sander, Enrique Herrero Acero, Alessandro Pellis, Georg M. Guebitz

**Affiliations:** ^1^Austrian Centre of Industrial BiotechnologyTulln, Austria; ^2^Institute of Environmental Biotechnology, University of Natural Resources and Life Sciences ViennaTulln, Austria; ^3^Dipartimento di Scienze Chimiche, Universita degli Studi di Napoli Federico IINaples, Italy; ^4^Institute of Biogeochemistry and Pollutant Dynamics, ETH ZurichZurich, Switzerland

**Keywords:** cutinase, *Thermobifida cellulosilytica*, *Pichia pastoris*, poly(3-hydroxybutyrate-co-3-hydroxyvalerate), poly(butylene succinate), poly(ethylene terephthalate), enzymatic hydrolysis, aliphatic polyesters

## Abstract

To study hydrolysis of aromatic and aliphatic polyesters cutinase 1 from *Thermobifida cellulosilytica* (Thc_Cut1) was expressed in *P. pastoris*. No significant differences between the expression of native Thc_Cut1 and of two glycosylation site knock out mutants (Thc_Cut1_koAsn and Thc_Cut1_koST) concerning the total extracellular protein concentration and volumetric activity were observed. Hydrolysis of poly(ethylene terephthalate) (PET) was shown for all three enzymes based on quantification of released products by HPLC and similar concentrations of released terephthalic acid (TPA) and mono(2-hydroxyethyl) terephthalate (MHET) were detected for all enzymes. Both tested aliphatic polyesters poly(butylene succinate) (PBS) and poly(3-hydroxybutyrate-co-3-hydroxyvalerate) (PHBV) were hydrolyzed by Thc_Cut1 and Thc_Cut1_koST, although PBS was hydrolyzed to significantly higher extent than PHBV. These findings were also confirmed *via* quartz crystal microbalance (QCM) analysis; for PHBV only a small mass change was observed while the mass of PBS thin films decreased by 93% upon enzymatic hydrolysis with Thc_Cut1. Although both enzymes led to similar concentrations of released products upon hydrolysis of PET and PHBV, Thc_Cut1_koST was found to be significantly more active on PBS than the native Thc_Cut1. Hydrolysis of PBS films by Thc_Cut1 and Thc_Cut1_koST was followed by weight loss and scanning electron microscopy (SEM). Within 96 h of hydrolysis up to 92 and 41% of weight loss were detected with Thc_Cut1_koST and Thc_Cut1, respectively. Furthermore, SEM characterization of PBS films clearly showed that enzyme tretment resulted in morphological changes of the film surface.

## Introduction

Plastic materials are ubiquitous in our daily life and although the annual European production is in a steady state since a decade, the global production is constantly increasing^1^. Most conventional plastics such as polyethylene, polypropylene, polystyrene, poly(vinyl chloride), and poly(ethylene terephthalate) (PET) are fully petrol-based and not biodegradable. The release of those plastic materials into the environment and their subsequent accumulation poses environmental risks and negatively impacts ecosystems, including the extreme consequences of plastic patch formation in rivers and oceans (Eriksen et al., 2014; Lechner et al., 2014). Considerable effort has been directed toward implementing bio-based plastics as environmentally-friendly alternatives to the traditionally petrol-derived materials. In particular, the substitution of polyesters such as PET and polybutyrate adipate terephthalate (PBAT) seems to be imminent since several market-leading companies are focusing their investigations on production of monomers derived from renewable biomass. Recent innovations also allow the biotechnological production of bio-based monomers from renewable carbon, enabling the replacement of petrochemical building blocks (Pellis et al., 2016c,d). These bio-based building blocks can be either produced by microbial conversions of various feedstocks or with combined biotechnological-chemical pathways that lead to various monomers such as 1,4-butanediol and adipic acid (used for the production of PBAT) (Harmsen et al., 2014). Fermentation of sugars or various other feedstocks, including lignocellulose (Pinazo et al., 2015), can also be used to obtain succinic and lactic acid for the production of poly(butylene succinate) (PBS) and poly(lactic acid) respectively. Poly(hydroxyalkanoates) (PHAs) on the other hand are directly produced by natural or engineered microorganisms. Mulch films are the most common and highly consumed plastic products in agriculture and their widespread use has led to an increase in environmental wastes. Therefore, commercially available mulch films are usually made of biodegradable plastics, with PBS as the main component (Koitabashi et al., 2012). In recent years there has been conservable interest in the substitution of PET with plant-derived poly(ethylene furanoate) (PEF) (Pellis et al., 2016b). The monomers for PEF (2,5-furandicarboxylic acid and ethylene glycol) can be 100% produced from renewable feedstocks (Pellis et al., 2016d).

The potential of enzymes for degradation of polymer building blocks has been studied by several groups. Various enzymes belonging to the cutinase family were reported to hydrolyze PET, the most used polyester (Yoon et al., 2002; Vertommen et al., 2005; Herzog et al., 2006; Heumann et al., 2006; Donelli et al., 2009; Herrero Acero et al., 2011; Ribitsch et al., 2011, 2012; Kanelli et al., 2015). Moreover, reports on the biocatalyzed hydrolysis of poly(lactic acid) (Pellis et al., 2015, in press; Ortner et al., 2017), poly(butylene succinate) (Hu et al., 2016) and poly(ethylene furanoate) (Pellis et al., 2016b) using similar biocatalysts were also described and certify the importance of such processes in the optics of a sustainable development (Clark et al., 2016; Pellis et al., 2016c). Despite the high industrial potential reported for cutinases from *Thermobifida* spp., these enzymes have usually been obtained by intracellular recombinant expression in *E. coli* (Herrero Acero et al., 2011; Su et al., 2013; Roth et al., 2014; Then et al., 2016), an approach that hampers the scale-up of the production process. Lately the methylotrophic yeast *P. pastoris* gained increasing interest as expression system for recombinant proteins for basic research as well as for industrial applications as shown by the number of filed patents (Bollok et al., 2009). In addition to the ability of *P. pastoris* to perform post-translational modifications one of the main advantages is that the recombinant proteins can often be secreted at high concentrations while maintaining their correct folding and activity (Cregg et al., 1993; Cereghino and Cregg, 2000; Ahmad et al., 2014; Hu et al., 2016). Furthermore, this host usually allows a simple production scale-up by changing from shaking flaks expressions to (fed-batch) fermenters (Schilling et al., 2002; Johnson et al., 2003; Zhao et al., 2008). Several commercial proteins are produced in *P. pastoris*, including recombinant *Tritirachium album* Proteinase K (Thermo Scientific, Waltham, MA, USA), Trypsin (Roche Applied Science, Germany), and nitrate reductase (The Nitrate Elimination Co., Lake Linden, MI, USA; Ahmad et al., 2014). In the past, successful expressions of cutinases from *Fusarium solani* (Kwon et al., 2009; Hu et al., 2016), *Alternaria brassicicola* (Koschorreck et al., 2010), *Glomerella cingulata* (Seman et al., 2014), and *Trichoderma harzianum* (Rubio et al., 2008) in *P. pastoris* have been reported. In this study, cutinase 1 from *Thermobifida cellulosilytica* (Thc_Cut1) as well as two glycosylation knock out mutants (Thc_Cut1_ko) were cloned and overexpressed in *P. pastoris* and screened for their ability to hydrolyze the aromatic polyester (PET) and the aliphatic polyesters [Poly(3-hydroxybutyrate-co-3-hydroxyvalerate) (PHBV) and PBS].

## Materials and methods

### Chemicals and reagents

Restriction enzymes, antarctic phosphatase, T4 DNA ligase as well as Endo H_f_ were obtained from New England Biolabs (USA). Synthetic genes of *P. pastoris* codon optimized Thc_Cut1 and glycosylation site knock out mutants (Thc_Cut1_ko_Asn and Thc_Cut1_ko_ST) cloned into pMK-T were ordered from GeneArt (Germany). Pro-Q® Emerald 300 Glycoprotein Gel and Blot Stain Kit (P21857), CandyCane glycoprotein molecular weight standard (C21852) as well as *P. pastoris* KM71H strain and expression vector pPICZαB were acquired from ThermoFisher Scientific (USA). *E. coli* XL-10 cells were purchased from Agilent (USA). PureYield™ Plasmid Midiprep System, SV Gel and PCR Clean-Up System Kits and Mini-PROTEAN® TGX (Stain-Free™) Precast Gels were obtained from Promega (Germany) or BioRad (USA), respectively. Peptone, Yeast extract and DifCo yeast nitrogen base were purchased from Becton Dickinson (USA) and Zeocin™ was obtained from Eubio (Austria). All other chemicals were of the highest available purity and ordered from Sigma-Aldrich. PET powder obtained from still water bottle from Cristaline® was kindly provided by Carbios (St-Beauzire, France) and was previously characterized (Gamerith et al., in press). PHBV was purchased from Metabolix while PBS was purchased from Sigma-Aldrich. The PBS material used for quartz crystal microbalance (QCM) experiments was obtained from BASF and the physicochemical properties of this polyester were previously reported (Zumstein et al., 2016).

### Designing of Thc_Cut1 glycosylation site knock out mutants

Using NetNGlyc 1.0 server (Technical University of Denmark) five possible N-glycosylation sites were predicted in the native Thc_Cut1 sequence (GenBank accession no. ADV92526.1). Asparagine (Asn) at amino acid position 10 is directly followed by a proline which makes glycosylation unlikely due to conformational constraints. Also for Asn at position 233 the glycosylation potential was lower compared to the other potential glycosylation sites according to the prediction. Therefore, the three glycosylation sites at Asn 29, Asn 49, and Asn 161 were knocked out by changing the nucleotide sequence accordingly, resulting in two triple knockout mutants (for details see Table 1). Synthetic genes of the designed glycosylation site knock out mutants (Thc_Cut1_ko) cloned into pMK-T were ordered from GeneArt.

**Table 1:** **Detailed design of Thc_Cut1 glycosylation site triple knock out mutants (Thc_Cut1_ko)**.

**Name**	**AA mutations**			**Nucleotide mutations**	
	**Position**	**From**	**To**	**From**	**To**
Thc_Cut1_ko_Asn	29	Asn	Asp	AAC	GAC
	49	Asn	Asp	AAC	GAC
	161	Asn	Asp	AAC	GAC
Thc_Cut1_ko_ST	31	Ser	Ala	TCT	GCA
	51	Thr	Ala	ACT	GCA
	163	Ser	Ala	TCC	GCA

### General recombinant DNA techniques

All general recombinant DNA techniques described in this work were performed following previously reported standard protocols (Sambrook et al., 1989). Digestion of cloning vector (pMK-T) and expression vector (pPICZαB) were performed with *Not*I Hf and *Xho*I, dephosphorylation was performed by antarctic phosphatase and T4 DNA ligase was used for ligation according to manufacturer's protocols (New England Biolabs). Plasmids and DNA fragments were purified by PureYield™ Plasmid Midiprep System Kit or Wizard® SV Gel and PCR Clean-Up System Kit. After transformation of *E. coli* XL-10 cells and plasmid purification pPICZαB_Thc_Cut1 and pPICZαB_Thc_Cut1_ko constructs were sequenced by LGC Genomics in order to confirm the DNA sequence.

### Transformation into *P. pastoris* KM71H and screening of transformants

Eighty Milliliter 80 μL of electrocompetent *P. pastoris* KM71H cells were transformed with *Sac*I-Hf linearized pPICZαB_Thc_Cut1 or pPICZαB_Thc_Cut1_ko by electroporation (MikroPulser™, Bio-Rad) according to the manual instructions. Transformed cells were spread on yeast extract peptone dextrose sorbitol medium agar plates [YPDS, 1% (*w/v*) yeast extract, 2% (*w/v*) peptone, 2% (*w/v*) glucose, 1 M sorbitol, 2% (*w/v*) agar] containing 0.1 mg/mL Zeocin™ and incubated at 28°C for 3–5 days. Transformants were cultivated in YPD medium in 96-deep-well-plates and screened for multi-copy integrants on YPD agar plates [1% (*w/v*) yeast extract, 2% (*w/v*) peptone, 2% (*w/v*) glucose, 2% (*w/v*) agar] containing 0.1–2 mg/mL Zeocin™. Stock cultures of selected clones were stored at −80°C.

### *P. pastoris* shaking flask fermentation

For enzyme production, 1 L baffled shaking flasks containing 250 mL of buffered glycerol complex medium [BMGY; 1% (*w/v*) yeast extract, 2% (*w/v*) peptone, 1% (v/*v*) glycerol, 3.4% (*w/v*) yeast nitrogen base, 4 × 10^−5^% biotin, 100 mM potassium phosphate buffer pH 6.0] were inoculated with *P. pastoris* KM71H transformants and incubated at 28°C and 150 rpm for approximately 16–18 h. Cells were harvested by centrifugation (3,000 × g, 8 min, 22°C) and the cell pellet was re-suspended in one-tenth of the original volume (75 mL culture volume in 300 mL shaking flasks). Enzyme expression was induced by the addition of methanol to a final concentration of 1% (*v/v*). Methanol was added twice daily to a final concentration of 1% (*v/v*) to sustain the induction. During fermentation, samples were collected by centrifugation (14,000 rpm, 5 min, 22°C) and supernatants were stored at −20°C until further use. After up to 120 h of enzyme expression, cells were harvested by centrifugation (4,500 rpm, 4°C, 20 min) and the supernatant was stored at −20°C until protein purification.

### Immobilized metal ion affinity chromatography for enzyme purification

The enzyme purification from the fermentation supernatants was performed via affinity chromatography (ÄKTA purifier, GE Healthcare) using HisTrap™ excel 5 mL columns (GE Healthcare). After sample loading (flow rate 2 mL/min) the column was washed with 7 column volumes (CV) of equilibration buffer (20 mM NaH_2_PO_4_, 500 mM NaCl, pH 7.4) followed by 3 CV of 1% elution buffer (20 mM NaH_2_PO_4_, 500 mM NaCl, 500 mM imidazole, pH 7.4). The enzyme was eluted with 45% elution buffer for 6 CV. Finally the column was washed with 100% elution buffer for 3 CV and stored in a 20% ethanol solution. Proteins were detected at 280 nm. SDS-PAGE analysis of purification fractions was performed in order to confirm the presence of Thc_Cut1 or Thc_Cut1_ko in the pooled fractions. PD-10 columns (Sephadex™ G-25 Medium, GE Healthcare) were used to exchange the buffer to 100 mM KH_2_PO_4_/K_2_HPO_4_ pH 7.0 before storage of purified proteins at −20°C until further usage.

### Expression analysis and enzyme characterization

#### SDS-PAGE and glycostain analysis

SDS-PAGE of fermentation supernatant samples withdrawn at different time points was performed according to standard conditions (Laemmli, 1970). After staining with Coomassie Brilliant Blue R-250, SDS PAGE gels were imaged using a ChemiDoc (Chemidoc™ MP Imaging System, Bio-Rad). Stain free SDS-PAGE gels were directly visualized using a ChemiDoc (Chemidoc™ MP Imaging System, Bio-Rad) without further treatment. Deglycosylation of Thc_Cut1 and Thc_Cut1_ko mutants was performed using Endo H_f_ according to manufacturer's instructions (New England Biolabs). Glycostain gels were prepared according to Pro-Q® Emerald 300 Glycoprotein Gel and Blot Stain Kit manual and detected by G-Box or hand-held UV lamp at 300 nm.

#### Protein analysis

Total protein concentrations in the fermentation supernatants (from different time points) as well as protein concentrations of purified enzymes were determined using the Bradford assay (BioRad) according to manual instructions and using bovine serum albumin (BSA) as standard.

#### Esterase activity assay

Esterase activity of fermentation supernatants (from different time points) and of purified enzymes was measured using *p*-nitrophenyl butyrate (pNPB) as soluble substrate according to Gamerith et al. using the experimentally determined extinction coefficient (ε = 9.7 mL μmol^−1^cm^−1^) (Gamerith et al., in press).

### Enzymatic hydrolysis of polyester substrates

For hydrolysis reactions, 50 mg of PET powder or 5 mg of aliphatic polyester powders (PHBV, PBS) were weighed and incubated with 5 μM of enzyme (Thc_Cut1 or Thc_Cut1_koST) diluted in a final volume of 1 mL in KH_2_PO_4_/K_2_HPO_4_ (1 M, pH 8.0). In case of PBS films, pieces of 0.5 × 1.0 cm were cut and washed in three serial steps (5 g/L Triton X-100, 100 mM Na_2_CO_3_, and ddH_2_O; each for 30 min at 50°C and 100 rpm) prior to hydrolysis reactions in order to remove possible surface contaminations (Pellis et al., 2015, in press; Gamerith et al., 2016). Incubations were performed in 2 mL tubes at 100 rpm and 65°C for different time frames. The released acids, alcohols and oligomers, namely: terephthalic acid (TPA) and mono(2-hydroxyethyl) terephthalate (MHET) for PET; succinic acid (SA) and 1,4-butanediol (BDO) for PBS and 3-hydroxybutyric acid (3-HBA) for PHBV were analyzed by HPLC using either a diode array detector (DAD) or a refractive index detector (RI). As a blank, polyester substrates were incubated in KH_2_PO_4_/K_2_HPO_4_ (1 M, pH 8.0) without enzyme. Enzyme blanks were also performed by incubating 5 μM solutions of enzymes in KH_2_PO_4_/K_2_HPO_4_ (1 M, pH 8.0) without polyester substrate. All hydrolysis experiments were performed in triplicates.

### Analysis of soluble monomers and oligomers released by high performance liquid chromatography (HPLC-DAD or HPLC-RI)

#### HPLC-DAD detection of TPA and MHET

HPLC analysis of released products upon enzymatic hydrolysis of PET was performed as recently described by Gamerith et al. (in press). Briefly, after enzyme treatment of polyester powders the enzyme was precipitated with ice-cold methanol. After acidification to pH 3.5, samples were centrifuged (Hettich MIKRO 200 R, Tuttlingen, Germany) at 14,000 rpm at 0°C for 15 min, filtered (0.45 μm nylon) and transferred to HPLC vials. For HPLC (Agilent Technologies, 1260 Infinity) analysis, a reversed phase column C18 (YMC 30, 250 × 4.6 mm ID, S-5 μm) was used. Analysis was carried out with constant 10% 0.01 N formic acid and starting with 85% water and 5% methanol, gradual (1 min) to 10% methanol, gradual (to 8 min) to 50% methanol and gradual (to 10 min) to 90% methanol, back to starting position with a 7 min post run. The flow rate was set to 0.85 mL min^−1^ and the column was maintained at a temperature of 40°C. The injection volume was set to 10 μL. Detection of the analytes was performed with a photodiode array detector (Agilent Technologies, 1290 Infinity II) at a wavelength of 241 nm. Standards of TPA and bis(hydroxyethyl)terephthalate (BHET) were prepared in KH_2_PO_4_/K_2_HPO_4_ (1 M, pH 8.0) in a range of 0.005–0.5 mM and treated the same way as samples.

#### HPLC-RI detection of SA, BDO, and 3-HBA

HPLC-RI detection of released products from PHBV and PBS was performed as previously reported by Pellis et al. (2015). Briefly, hydrolysis samples were precipitated following the Carrez method and filtered through 0.45 μm Nylon filters (GVS, Indianapolis, USA). The analytes were separated by HPLC using refractive index detection (1100 series, Agilent Technologies, Palo Alto, CA) equipped with an ICSep-ION-300 column (Transgenomic Organic, San Jose, CA) of 300 mm by 7.8 mm and 7 μm particle diameter. Column temperature was maintained at 45°C. Samples (40 μL) were injected and separated by isocratic elution for 40 min at 0.325 mL min^−1^ in 0.005 M H_2_SO_4_ as the mobile phase. Standards of SA, BDO and 3-HBA were prepared in KH_2_PO_4_/K_2_HPO_4_ (1 M, pH 8.0) in a range of 0.5–100 mM and treated the same way as samples.

### Enzymatic hydrolysis measurements using a quartz crystal microbalance (QCM)

The hydrolysis of spin-coated PHBV and PBS thin films by Thc_Cut1 was measured by QCM as previously reported (Zumstein et al., 2016). In brief, we spin coated thin films from chloroform solutions containing the respective polyester (concentration: 0.5% w/w) onto the surfaces of gold-coated QCM sensors. After air-drying the sensors, they were incubated in a buffered solution [3 mM tris(hydroxymethyl)-aminomethane, 10 mM potassium chloride, pH 7.0] for 14 h. The sensors were subsequently mounted into the flow cells of a QCM instrument (model E4, Q-sense) and rinsed with buffered solution of the same composition at a volumetric flow rate of 20 μL/min and a temperature of 40°C. Upon attaining stable resonance frequencies of the fundamental tone and several oscillation overtones, we switched to delivering solutions that contained Thc_Cut1 (2.07 μg/mL) but otherwise were identical in pH and ionic composition to the solutions used for equilibration. We subsequently monitored changes in the resonance frequencies over the course of the hydrolysis experiment and related these frequency changes to adlayer mass changes using the Sauerbrey equation. We used the fifth overtone of the oscillation for calculations and data plotting. To measure the fraction of coated polyester dry mass that was removed over the course of the hydrolysis experiment, we measured the resonance frequency of each sensor in air after the experiment as well as before and after the initial polyester spin coating step. We note that Thc_Cut1 used in QCM experiments was expressed in *E. coli*.

### Scanning electron microscopy (SEM)

PBS films morphology was qualitatively assessed through scanning electron microscopy (SEM). Control PBS (without any enzymatic treatment) and enzymatically hydrolyzed films (after 24, 48, 72, and 96 h) were surface characterized. All SEM images were acquired collecting secondary electrons on a Hitachi 3030TM (Metrohm INULA GmbH, Austria) working at EDX acceleration voltage.

## Results and discussion

### Glycosylation site knock out mutant design, vector construction, and transformation in *P. pastoris*

**Figure 1:**
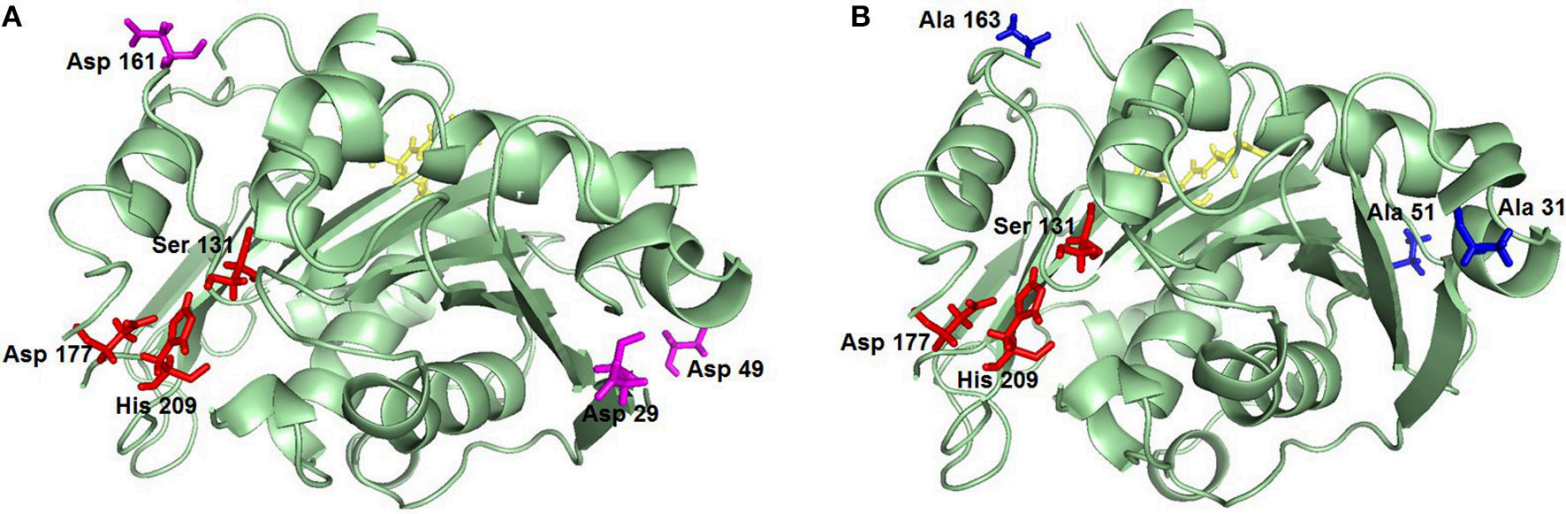
**Representation of Thc_Cut1 glycosylation site knockout mutants. (A)** Thc_Cut1_koAsn, **(B)** Thc_Cut1_koST; mutated AA are in pink **(A)** and blue **(B)**, active site residues are in red, ALE linker sequence for 6× His-Tag is shown in yellow.

In its natural hosts or when heterologously expressed in *E. coli* (Herrero Acero et al., 2011; Su et al., 2013; Roth et al., 2014; Then et al., 2016) *Thermobifida* spp. cutinases are not glycosylated. In contrast, expression in *P. pastoris* may lead to glycosylation which can have positive effects such as increased stability, as previously shown for a *Thermobifida* xylanase (Zhao et al., 2015), human aquaporin 10 (Öberg et al., 2011) and *Rhizopus chinensis* lipase (Yang et al., 2015). Shirke et al. also recently reported that glycosylation stabilizes a *P. pastoris*-expressed cutinase from *Thiellavia terrestris* by inhibiting its thermal aggregation (Shirke et al., 2017). On the other hand, glycosylation may also have negative effects, as shown for example for a lipase from *Rhizomucor miehei* which had decreased activity upon N-glycosylation (Liu et al., 2014). Therefore, it was important to investigate the influence of glycosylation on the activity and stability of Thc_Cut1 when expressed in *P. pastoris*. Hence, two glycosylation site triple knock out mutants were designed (Figure 1). The recombinant pPICZαB_Thc_Cut1 and pPICZαB_Thc_Cut1_ko plasmids contained the codon optimized gene of wild type or mutated Thc_Cut1, the methanol inducible alcoholoxidase 1 promoter (AOX1), the *S. cerevisiae* α-factor secretion signal, a C-terminal 6x His-Tag and a transcription termination signal. The tightly regulated AOX1 promoter holds advantages for overexpression of proteins since cells are not stressed by the accumulation of recombinant protein during growth phase. Even the production of proteins that are toxic to *P. pastoris* is possible by uncoupling the growth from the production phase (Ahmad et al., 2014). The most commonly employed method of generating multi-copy expression strains in *P. pastoris* is based on direct screening of transformants on agar plates containing increasing concentrations of antibiotics (e.g., 0.1–2 mg/mL of Zeocin™) (Ahmad et al., 2014). After successful transformation by electroporation the selection of Thc_Cut1 and Thc_Cut1_ko transformants yielded clones that might contain multi-copy integrations as shown by growth on 2 mg/mL Zeocin™. A direct correlation between copy number and expression level has been shown especially for intracellular expression (Vassileva et al., 2001; Marx et al., 2009), but this direct correlation is not necessarily valid for secreted proteins (Marx et al., 2009).

### Analysis of cutinase expression in shaking-flasks

**Figure 2:**
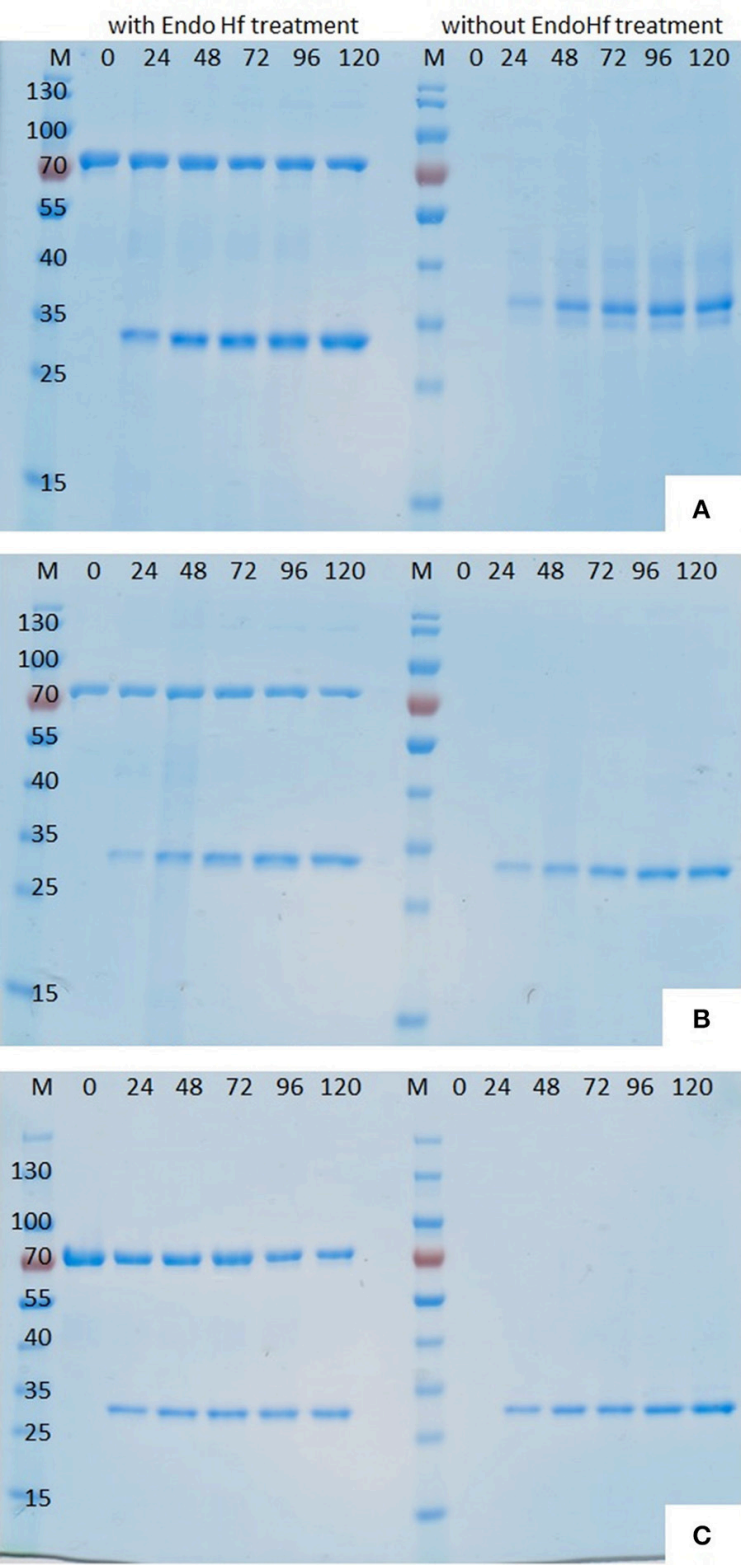
**SDS-PAGE (12%) of ***P. pastoris*** fermentation supernatant samples withdrawn at different time points after induction. (A)** Thc_Cut1, **(B)** Thc_Cut1_koAsn, **(C)** Thc_Cut1_ko_ST; M = peqGold protein marker IV, #= time after methanol induction in h, each sample set was once loaded after treatment with Endo Hf (left) and once without Endo Hf treatment (right).

After successful transformation and screening on high Zeocin™ YPD agar plates the best growing *P. pastoris* KM71H transformants of each enzyme were chosen for enzyme production in shaking flasks. Enzyme expression was induced by the addition of methanol and during fermentation several supernatant samples were collected by centrifugation. Analysis of these supernatant samples drawn at different time points during shaking flask fermentations by SDS-PAGE clearly showed that methanol induction successfully stimulated the expression of cutinases (Figure 2).

**Figure 3:**
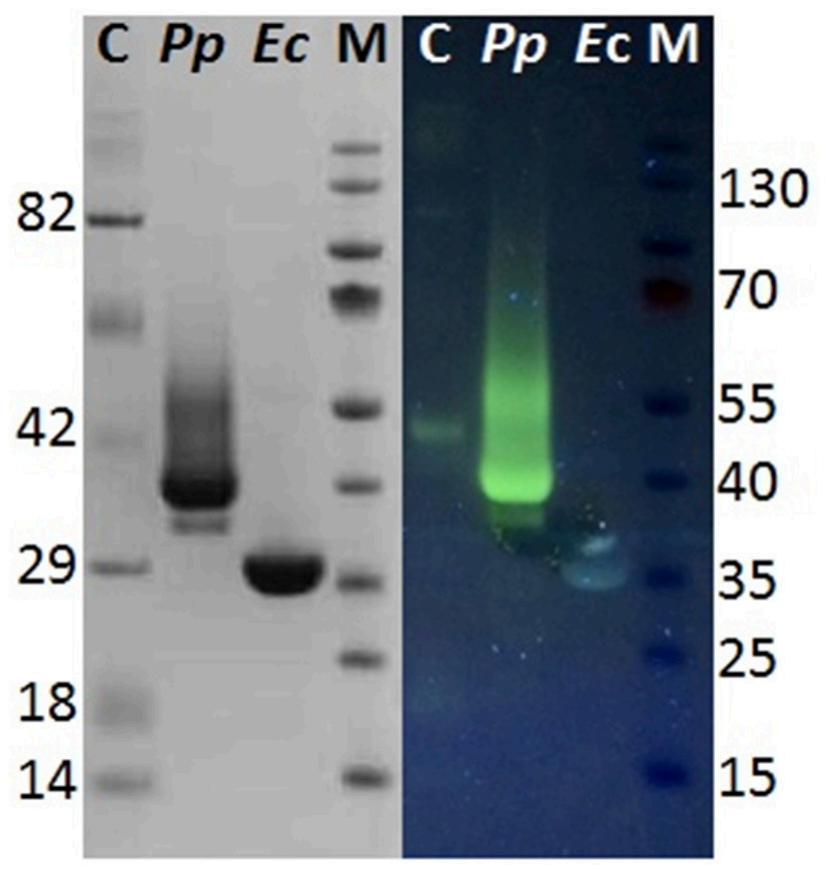
**Glycostain gel analysis of ***P. pastoris*** and ***E. coli*** expressed Thc_Cut1**. Left, SDS PAGE gel; Right, Glycostained gel; same samples were applied on both gels; C, Candy cane protein marker; M, peqGold protein marker IV; Pp, *P. pastoris* expressed Thc_Cut1; Ec, *E. coli* expressed Thc_Cut1.

Although hyperglycosylation of heterologous proteins is more common in *S. cerevisiae* (Grinna and Tschopp, 1989), expression in *P. pastoris* can also lead to hyperglycosylation mainly attributed to N-mannosylation (Bretthauer and Castellino, 1999; Várnai et al., 2014). Glycosylation may affect the migration of the proteins on SDS-PAGE or, in case of heterogeneous glycosylation, may result in smears (Bretthauer and Castellino, 1999; Várnai et al., 2014). It was previously reported that heterologous expression of cutinase CUTAB1 from *Alternaria brassiciola* in *P. pastoris* led to a single band on SDS-PAGE when applied as crude supernatant. However, when applied after purification, an additional band became more distinct. Since purified and Endo H_f_ deglycosylated CUTAB1 only showed one band, the two different bands were assigned to the glycosylated and non-glycosylated enzyme (Koschorreck et al., 2010). In our case Thc_Cut1 appeared as a distinctive band around 38 kDa (Figure 2A, right), indicating its high level of glycosylation, whereas Endo H_f_ deglycosylated Thc_Cut1 showed one clear band corresponding to the calculated mass of 29.4 kDa (Figure 2A, left; Herrero Acero et al., 2011). The protein band around 70 kDa corresponds to Endo H_f_ used for deglycosylation. On the contrary, Thc_Cut1_ko mutants showed a clear band around 29 kDa with or without Endo H_f_ treatment suggesting that the glycosylation sites were successfully knocked out in both mutants (Figures 2B,C). These results were confirmed by staining SDS PAGE gels with Pro-Q® Emerald 300 glycoprotein stain, which creates a bright green-fluorescent signal on glycoproteins. For direct comparison of Commassie- and Glyco-staining, the same samples were loaded on two SDS PAGE gels whereas only one gel was glycostained afterwards. Glycostained gels showed clearly fluorescent bands of purified native Thc_Cut1 expressed by *P. pastoris*, whereas no fluorescent bands were detected for Thc_Cut1 expressed by *E. coli* (Gamerith et al., in press; Figure 3) or for Endo H_f_ deglycosylated Thc_Cut1 expressed by *P. pastoris* (see Figure S1).

**Figure 4:**
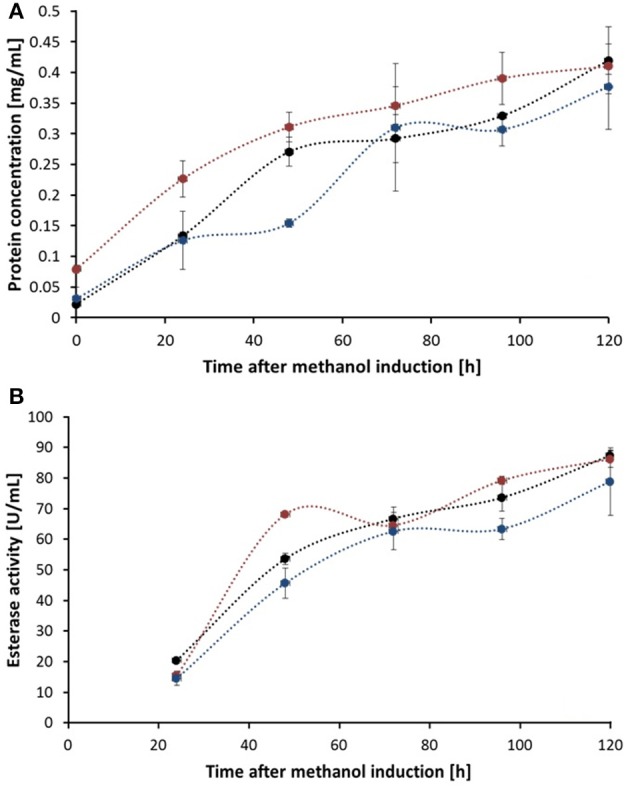
**Total protein concentration (A) and esterase activity (B) of fermentation supernatant samples of different time points after methanol induction**. Thc_Cut1 (black line), Thc_Cut1_ko_Asn (blue line), and Thc_Cut1_ko_ST (red line). Time point 0 h indicates the start of methanol feed. Error bars show standard deviations of triplicate measurements.

Although all fermentation supernatant samples from different time points during expression of Thc_Cut1_ko mutants showed high unspecific fluorescent signals, no specific bands corresponding to Thc_Cut1 were detected, suggesting a high background noise of the medium (see Figure S2 for Thc_Cut1_ko_ST). Also purified glycosylation site knockout mutants did not show any fluorescent bands, verifying the successful knock out of all glycosylation sites (Figure S2, last lane for purified Thc_Cut1_ko_ST as example). Post-translational glycosylation processes might have an influence on the expression level due to their time- and energy-demand. Furthermore, also dissolved oxygen concentrations and careful control of the methanol levels are crucial for a high expression of recombinant proteins in *P. pastoris* (Seman et al., 2014). Methanol might not only have toxic effects for the cells but, as a highly flammable and hazardous substance it is also problematic for large-scale applications (Ahmad et al., 2014). Nevertheless, studies on methanol-inducible promoters, including AOX1, have shown that protein expression can also be achieved without methanol induction by constitutive co-expression of positively acting transcription factor Prm1p from either of the GAP, TEF, or PGK promoters (Takagi et al., 2012). In agreement with SDS-PAGE analysis also an increase of total extracellular protein concentration, determined by Bradford assay (BioRad) and (BSA) as standard, as well as an increase in volumetric esterase activity on *p*NPB as substrate was detected in fermentation supernatants over time (Figures 4A,B, respectively). Within 24 h from the methanol addition, induction resulted in a clear increase in the total extracellular protein concentration. Interestingly, no significant differences between the expression of native Thc_Cut1 and Thc_Cut1_ko mutants concerning the total extracellular protein concentration were observed. Furthermore, the volumetric activity of all enzyme variants on *p*NPB was of the same order of magnitude.

Heterologous expression of Thc_Cut1 and Thc_Cut1_ko mutants in *P. pastoris* resulted in about 400 ± 20 mg total extracellular protein per liter in shaking flasks without optimization of culture conditions. These results are comparable to the previously reported expression level of *F. solani* cutinase in *P. pastoris* of 340 mg extracellular protein per liter (Kwon et al., 2009). In comparison to heterologous expression of CUTAB1 in *P. pastoris* by Koschorreck et al., yielding in 212 mg extracellular protein per liter, the expression level of Thc_Cut1 was almost doubled (Koschorreck et al., 2010). It is well-known that especially in the case of *P. pastoris* optimization of expression conditions in shake flasks or fed-batch fermenters can largely improve protein yields (Schilling et al., 2002; Zhao et al., 2008). However, the scope of this study was to demonstrate the general feasibility of Thc_Cut1 expression in *P. pastoris*.

### Immobilized metal ion affinity chromatography (IMAC) for enzyme purification

From the 70 mL crude supernatants of each enzyme 65 mL were loaded onto HisTrap™ excel columns resulting in 14 mL of purified and buffer exchanged enzymes with different concentrations and esterase activities (Table 2). Interestingly, around 75% of the native Thc_Cut1 and Thc_Cut1_koST could be recovered from the crude supernatant, whereas only around 34% of Thc_Cut1_koAsn could be purified. Protein peaks of Thc_Cut1 and Thc_Cut1_ko were detected at 280 nm and the presence of enzymes in the corresponding fractions was confirmed by SDS-PAGE analysis (see Figure S3 for example of Thc_Cut1 purification).

**Table 2:** **Production of various ThC-Cut1 variants in ***P. pastoris*****.

**Enzyme**	**Purification step**	**Total protein (mg)^a^**	**Total activity (U)^b^**	**Specific activity (U/mg)**	**Yield (%)^c^**
Thc_Cut1	Crude supernatant	27	5,700	210	100
	Purified and buffer exchanged	21	2,100	100	75
Thc_Cut1_koAsn	Crude supernatant	25	5,100	210	100
	Purified and buffer exchanged	8	1,432	170	34
Thc_Cut1_koST	Crude supernatant	27	5,600	210	100
	Purified and buffer exchanged	20	3,500	180	75

a *Protein concentration was determined as described in section Protein Analysis and calculated for total volume*.

b *Esterase activity was determined as described in section Esterase Activity Assay and calculated for total volume*.

c *Relative yield with protein content of crude supernatant set to 100%*.

Kwon et al. reported a negative effect of a C-terminal 6xHis tag on the cellular process for proper synthesis, folding, and secretion of *F. solani* cutinase in *P. pastoris* (Kwon et al., 2009). Similarly, also C-terminal fusion of small tags [such as FLAG-(Gly)5 and His-(Gly)5 tags] to the extracellular domain of human Fas ligand (hFasLECD) led to a failure in secretion of functional protein in *P. pastoris*, whereas the secretion of functional hFasLE CD was retained upon N-terminal tagging (Muraki, 2006). Nonetheless, since all cutinases used in this study had a C-terminal 6xHis tag, it was not possible to assess any effect of C-terminal tags within this study.

### Enzymatic hydrolysis of aromatic polyesters (PET)

**Figure 5:**
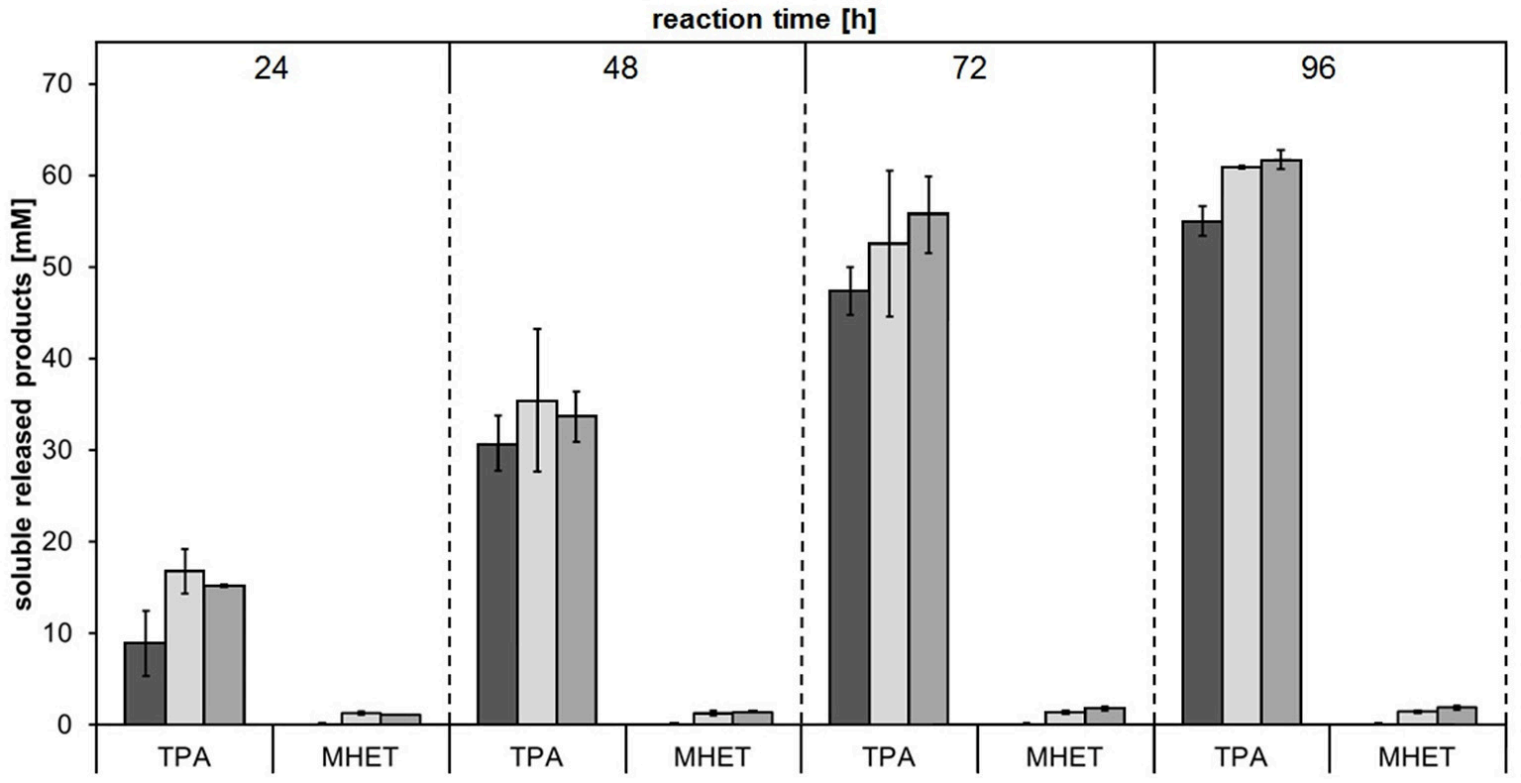
**Concentrations of soluble released products terephthalic acid (TPA) and mono(2-hydroxyethyl) terephthalate (MHET) upon enzymatic hydrolysis of PET powder by Thc_Cut1 (dark gray bars), Thc_Cut1_ko_Asn (light gray bars) and Thc_Cut1_ko_ST (middle gray bars)**. Time scan for 24, 48, 72, or 96 h was performed at 65 °C with 5 μM enzyme in 1 M KPi pH 8.0 with 50 mg/mL substrate at 100 rpm.

Several cutinases (Vertommen et al., 2005; Heumann et al., 2006; Donelli et al., 2009; Kanelli et al., 2015)—including *E. coli* expressed Thc_Cut1 (Herrero Acero et al., 2011)—have been found to hydrolyze PET. For this reason, this aromatic polyester was chosen as substrate for performing the first hydrolysis experiments with cutinases expressed in *P. pastoris* in order to confirm their activity. Besides the crystallinity of polyesters (Mochizuki and Hirmai, 1997; Vertommen et al., 2005; Herzog et al., 2006; Mueller, 2006; Brueckner et al., 2008; Tokiwa et al., 2009; Pellis et al., 2016a), also the incubation temperature is well-known to affect the enzymatic hydrolysis of polyesters, mainly by affecting the polymer chain mobility (Marten et al., 2003; Eberl et al., 2009). Incubation temperatures close to the glass transition temperature (*T*_g_) are suggested in order to promote enzymatic attack of polymers for degradation purposes (Mueller et al., 2005; Mueller, 2006; Kawai et al., 2014; Then et al., 2016) while *T* < *T*_g_ are instead suggested when the surface hydrophilization is desired (Pellis et al., 2015, in press; Ortner et al., 2017). We recently reported that, for short term reactions, higher incubation temperatures led to faster hydrolysis rates of PET by *E. coli* expressed Thc_Cut1 (Gamerith et al., in press) while for longer reaction times limited enzyme stability may counteract this effect. Furthermore, the ionic strength as well as the buffer choice were found to have a severe effect on enzymatic hydrolysis of PET by polyester hydrolases (Schmidt et al., 2016). High buffer concentrations might prevent the pH decrease of the incubating buffer during hydrolysis reactions due to the acidic released products (e.g., TPA). Hence, hydrolysis of a 24% crystalline PET powder with *P. pastoris* expressed Thc_Cut1, Thc_Cut1_ko_Asn and Thc_Cut1_ko_ST were performed at 65°C in 1M KH_2_PO_4_/K_2_HPO_4_ pH 8.0 and released products were quantified by HPLC-DAD (Figure 5). No significant differences between the hydrolysis efficiency of the two glycosylation site knock out mutants could be observed, but treatment with the knock out mutants resulted in slightly increased TPA levels compared to the native Thc_Cut1. Up to 62 mM released TPA were observed after 96 h of hydrolysis, corresponding to ~24% degradation of initial PET powder to soluble TPA. Compared to previously reported results for PET hydrolysis with Thc_Cut1 by Gamerith et al.—using incubation conditions of 100 mM KH_2_PO_4_/K_2_HPO_4_ pH 7.0 and 60°C (Gamerith et al., in press)—a combination of increased incubation temperature, higher pH and increased buffer concentration resulted in significantly higher hydrolysis rates of PET by Thc_Cut1 in this current study. This high degree of hydrolysis signifies a big step toward feasibility of enzymatic recycling of polyesters.

It is interesting to note that while the two glycosylation site knock out mutants showed very similar results with regards to both expression as well as PET hydrolysis rates, the purification yields of purified Thc_Cut1_ko_Asn were significantly lower compared to Thc_Cut1_ko_ST. Due to the highest productivity (and therefore scalability) for the expression, further enzyme selectivity studies on aliphatic polyesters were performed using the native Thc_Cut1 and Thc_Cut1_koST.

### Enzymatic hydrolysis of aliphatic polyesters (PHBV and PBS)

**Figure 6:**
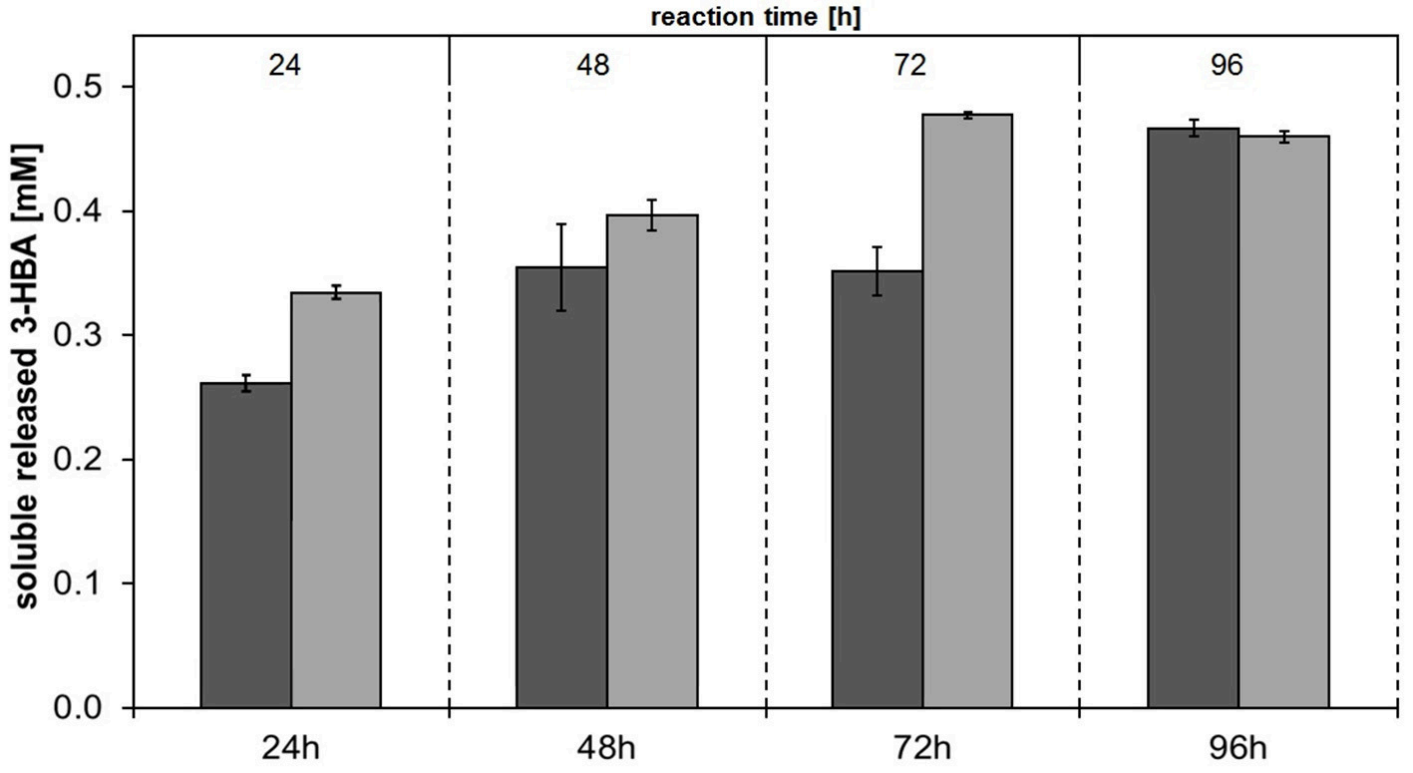
**Concentrations of soluble released 3-hydroxybutyric acid (3-HBA) upon enzymatic hydrolysis of PHBV powder by Thc_Cut1 (dark gray bars) and Thc_Cut1_ko_ST (middle gray bars)**. Time scan for 24, 48, 72, or 96 h was performed at 65 °C with 5 μM enzyme in 1 M KPi pH 8.0 with 5 mg/mL substrate at 100 rpm.

**Figure 7:**
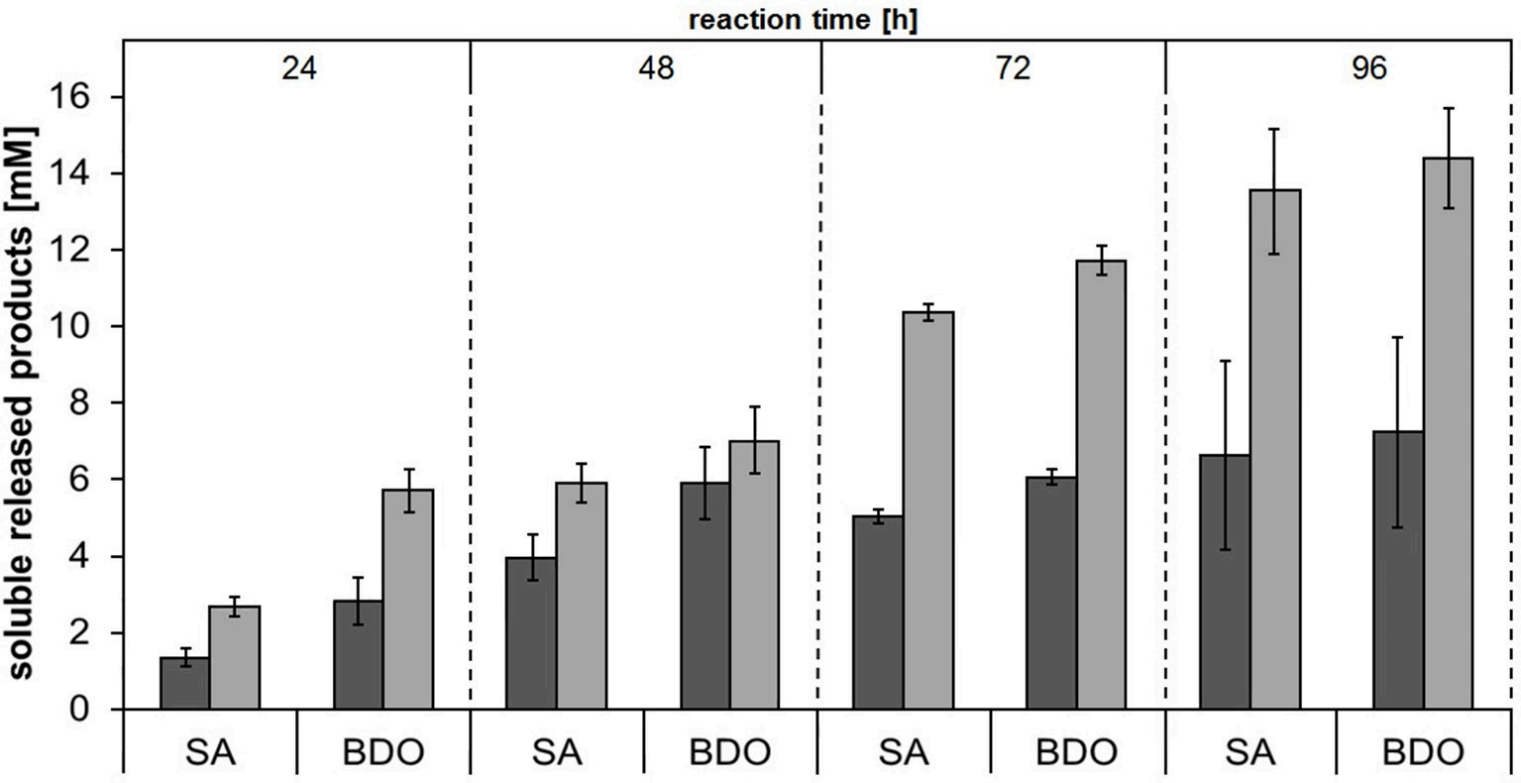
**Concentrations of soluble released products succinic acid (SA) and 1,4-butanediol (BDO) upon enzymatic hydrolysis of PBS powder by Thc_Cut1 (dark gray bars) and Thc_Cut1_ko_ST (middle gray bars)**. Time scan for 24, 48, 72 or 96 h was performed at 65°C with 5 μM enzyme in 1 M KPi pH 8.0 with 5 mg/mL substrate at 100 rpm.

In order to investigate the substrate specificities of Thc_Cut1 and Thc_Cut1_koST in more detail, hydrolysis experiments were performed using the aliphatic polyesters PHBV and PBS as substrates. Both aliphatic polyesters were successfully hydrolyzed by Thc_Cut1 and Thc_Cut1_koST, although to very different extents. Figures 6, 7 show the quantified concentrations of released products after up to 96 h enzymatic hydrolysis of PHBV and PBS powders, respectively. Interestingly, Thc_Cut1 and Thc_Cut1_koST reached similar levels of released 3-HBA (~0.5 mM) for the hydrolysis of PBHV, whereas for PBS, the released products were approximately twice as high for Thc_Cut1_koST as compared to native Thc_Cut1 (i.e., ~14 vs. ~7 mM SA and BDO). Due to different substrate concentrations (50 mg for PET vs. 5 mg for PBS and PHBV) the absolute values of released products seem lower compared to PET hydrolysis, but in fact the quantified SA and BDO concentrations correspond to ~24 and 48% degradation of initial PBS powder to soluble released products by Thc_Cut1 and Thc_Cut1_koST, respectively. This finding indicates that there was a remarkable influence of the glycosylation on the substrate specificity. Glycosylation may not only lead to increased stability and protection against proteolysis, but may also have a role on the catalytic activity. For several proteases it has been reported that glycosylation can alter their substrate recognition, their specificity and binding affinity, as well as the turnover rates. Moreover, glycans which are in the vicinity of the active site are more likely to influence the substrate binding (Goettig, 2016). Recently, we have demonstrated that both surface engineering as well as attachment of polymer binding modules or hydrophobins can dramatically influence sorption and thereby hydrolysis of polyesters (Herrero Acero et al., 2013; Ribitsch et al., 2013, 2015).

**Figure 8:**
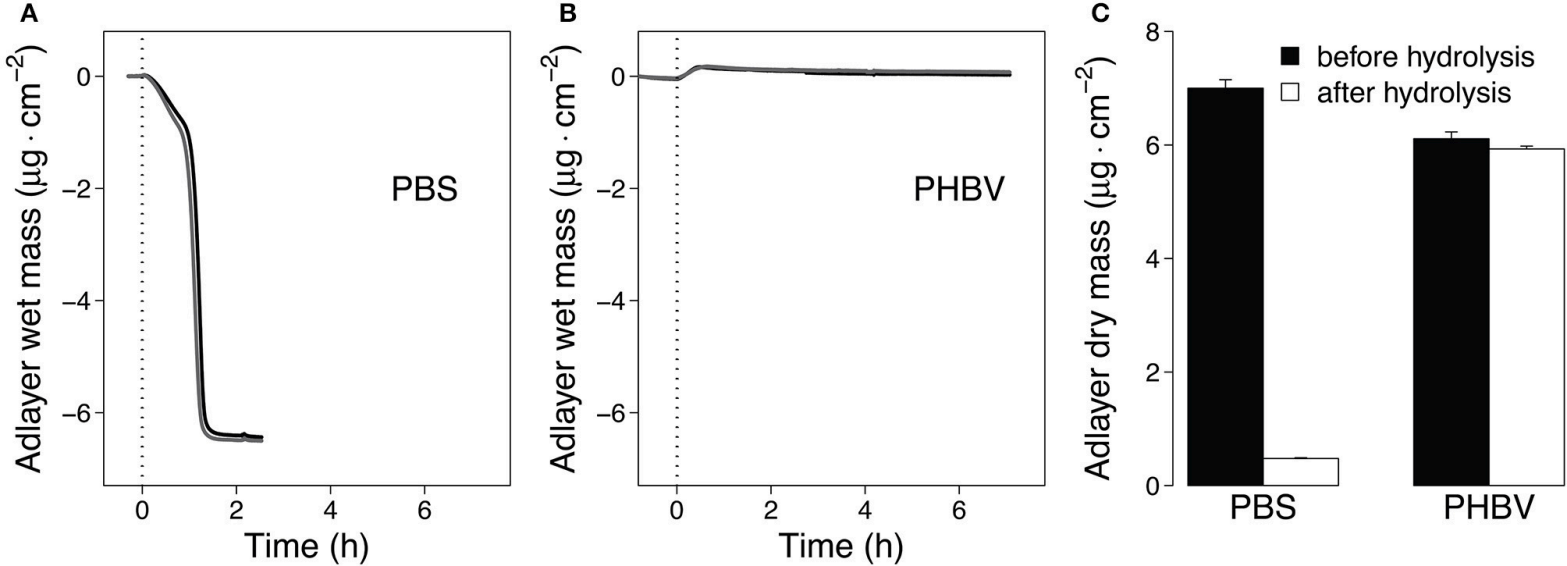
**Quartz Crystal Microbalance (QCM) measurements of hydrolysis of spin-coated PBS and PHBV films by Thc_cut1**. **(A,B)**. Progress curves of the polyester adlayer mass coated onto the QCM sensors during the hydrolysis of PBS **(A)** and PHBV **(B)**. At time *t* = 0 h we added Thc_cut1 to the buffer running over the films, as indicated by the vertical dashed lines in panels **(A,B)**. Black and gray lines represent duplicate experiments **(C)**. Dry masses of the sensor adlayers before and after the hydrolysis experiments. Hydrolysis experiments were performed at 40°C and pH 7.0 (3 mM Tris buffer).

To support faster Thc_Cut1-mediated hydrolysis of PBS than PHBV, we complemented the data above by QCM measurements. Previous studies showed that QCM can be used to study both adsorption of enzymes to the polyester surface and mass loss of polyester films due to enzymatic hydrolysis in real time (Ribitsch et al., 2013; Perz et al., 2015; Zumstein et al., 2016). Here, we monitored the mass change of spin-coated PBS and PBHV films during their hydrolysis by Thc_Cut1 (Figure 8). These measurements showed that the mass of the spin-coated PBS films rapidly decreased after the addition of Thc_Cut1 and that the adlayer mass reached stable final values within 1.5 h of the onset of Thc_Cut1 addition (Figure 8A; results of duplicate experiments). When Thc_Cut1 was added to PHBV films, we measured an initial adlayer mass increase that we ascribed to the adsorption of Thc_Cut1 to the film surface. Detection of this mass increase implies that PHBV hydrolysis was slow (in contrast to PBS). Slow PHBV hydrolysis was substantiated by the finding of slow and continuous decreases in the PHBV film mass over the subsequent hours of continuous exposure to Thc_Cut1 (Figure 8B). The differences in the mass decreases determined in the QCM-D measurements were consistent with the changes in the dry masses of the sensors, which we determined by measuring the adlayer masses of dried sensors before and after spin coating step and after the enzymatic hydrolysis experiment. These measurements revealed that 93 and 3% of the spin-coated PBS and PHBV masses, respectively, were removed during the hydrolysis experiments. We note that the Thc_Cut1 that was used for the QCM experiments was expressed in *E. coli*. This Thc_Cut1 variant is expected to have no glycosylation and to therefore show the same activity on polyesters as the Thc_Cut1_ko_ST variant that was expressed in *P. pastoris*, for which we showed the absence of glycosylation (Figure S2). In summary, the QCM-based analysis supported faster hydrolysis of PBS than PBHV by Thc_Cut1.

**Figure 9:**
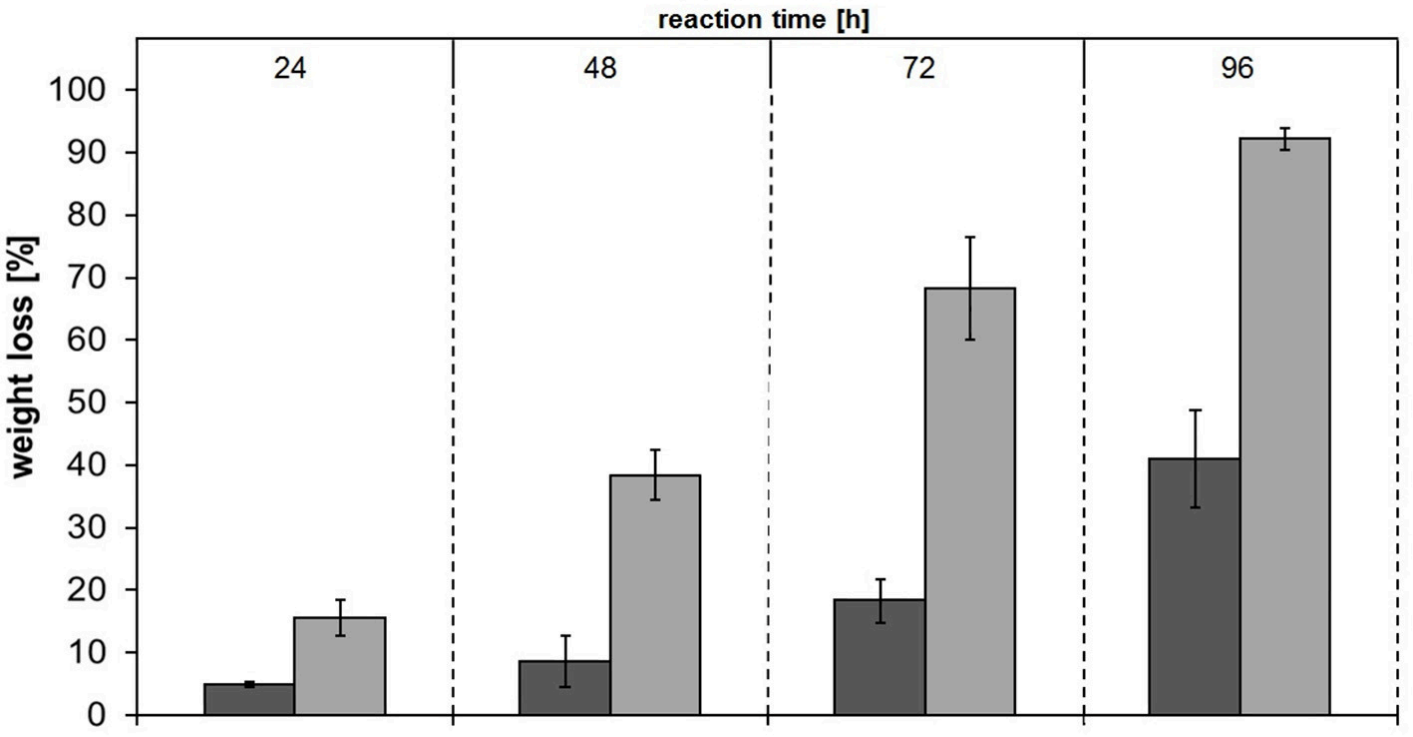
**Weight loss of PBS films upon enzymatic hydrolysis by Thc_Cut1 (dark gray bars) and Thc_Cut1_ko_ST (middle gray bars)**. Time scan for 24, 48, 72, or 96 h was performed at 65 °C with 5 μM enzyme in 1 M KPi pH 8.0 with 0.5 × 1.0 cm PBS films at 100 rpm

Among the tested polyesters, PBS was most extensively hydrolyzed and was therefore chosen for additional analyses. Hydrolysis of PBS films followed by weight loss and SEM analysis were performed. The concentrations of released products from PBS films showed the same trend as for PBS powder—Thc_Cut1_koST released more than double the amount of hydrolysis products compared to native Thc_Cut1 (~48–50 mM SA and BDO by Thc_Cut1_koST vs. ~12–15 mM SA and BDO by Thc_Cut1) (Figure 9 and Figure S4). These results are in accordance with the weight loss, which reached up to 92% with Thc_Cut1_koST and only around 41% with Thc_Cut1 within 96 h (see Figure 9). Hu et al. recently reported on complete degradation of PBS films by a recombinant cutinase from *Fusarium solani* within 6 h (Hu et al., 2016).

**Figure 10:**
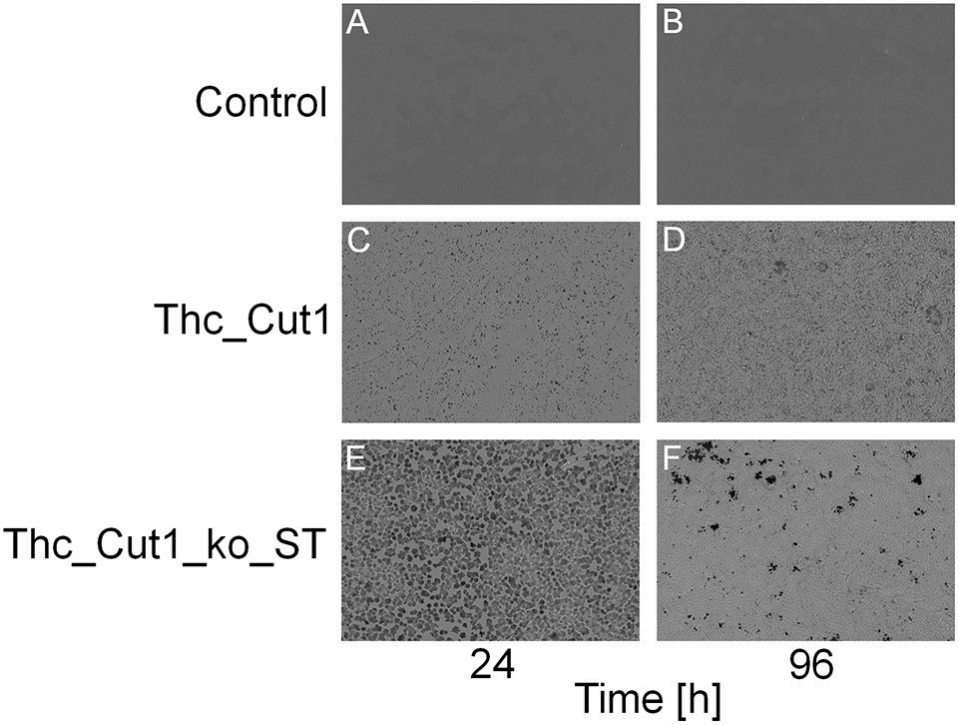
**SEM surface characterization of PBS films. (A,B)** control reactions (polymer film + buffer only); **(C,D)** Thc_Cut1-catalyzed hydrolysis (polymer film + Thc_Cut1 in buffer); **(E,F)** Thc_Cut1_ko_ST-catalyzed hydrolysis (polymer film + Thc_Cut1_ko_ST in buffer). All samples were measured after 24 (left column) and 96 h (right column) of treatment. It's clearly visible the increase of the hydrolysis reaction from 24 to 96 h **(C–F)** and the higher damage to the film by Thc_Cut1_ko_ST that yield, after 96 h to holes that go throughout the film **(E)**.

To complement the PBS hydrolysis data, an additional SEM characterization of the film surfaces was performed. Figure 10 shows clear changes in the morphology of PBS film surfaces caused by treatments with both native Thc_Cut1 and Thc_Cut1_koST (Figures 10C–F), while no detectable changes of the control samples occured (Figures 10A,B). Moreover, 24 h of enzymatic hydrolysis of the PBS films surface resulted in more surface erosion when using the ko mutant than the native Thc_Cut1 (Figure 10C vs. Figure 10E). After 96 h, the formation of “holes” throughout the polymeric sample is visible for the ko mutant (Figure 10F) while only an increased surface roughness was observed for the Thc_Cut1 treatment (Figure 10D).

## Conclusions

In this study, we demonstrated the general feasibility of expressing Thc_Cut1 and two glycosylation site knock out mutants, Thc_Cut1_koAsn and Thc_Cut1_koST, in *P. pastoris*. Furthermore, we have shown that Thc_Cut1 and Thc_Cut1_ko mutants hydrolyze aromatic (PET) and aliphatic (PHBV and PBS) polyester powders, although at very different rates as shown by HPLC quantification of released products. These findings were also confirmed by QCM measurements, which showed a 3.0% mass change for PHBV thin films and a 93.2% mass decrease for PBS thin films upon enzymatic hydrolysis with Thc_Cut1. The finding that treatment of PBS films with Thc_Cut1 and Thc_Cut1_koST resulted in large PBS weight losses and clear effects on film surface topography imaged by SEM confirm the potential of Thc_Cut1 and mutants for degradation of PBS films. Together with the high activity of Thc_Cut1 and Thc_Cut1_ko mutants on PET, this study provides a significant contribution toward enzymatic degradation of polyesters.

## Author contributions

CG, SG, and SZ expressed and purified the enzymes. DR designed the mutants. CG performed the PET hydrolysis experiments. MZ and MS conducted the QCM hydrolysis experiments and wrote the related sections of the manuscript. MV and AP performed the aliphatic polyesters hydrolysis of powders and films and the relative SEM images. CG, AP, EH, and GG wrote the manuscript.

### Conflict of interest statement

The authors declare that the research was conducted in the absence of any commercial or financial relationships that could be construed as a potential conflict of interest.

## Footnotes

^1^Plastics Europe Plastics—the Facts 2015, 2015 (Accessed May 17, 2016).
