# Supplementary material for: Enzymatic Degradation of Aromatic and Aliphatic Polyesters by P. pastoris Expressed Cutinase 1 from Thermobifida cellulosilytica
Source: Front Microbiol. 2017 May 24;8:938. doi: 10.3389/fmicb.2017.00938 (PMC5443175; doi:10.3389/fmicb.2017.00938)
Supplement: Supplementary file 1 [file DataSheet1.DOCX]

Supplementary Material

**Enzymatic Degradation of Aromatic and Aliphatic Polyesters by *P. pastoris* expressed Cutinase 1 from *Thermobifida cellulosilytica***

Caroline Gamerith^1^, Marco Vastano^2,3^, Sahar Masoumeh Ghorbanpour^2^, Sabine Zitzenbacher^1,a^, Doris Ribitsch^1,2^, Michael T. Zumstein^4^, Michael Sander^4^, Enrique Herrero Acero^1,b^, Alessandro Pellis^2,*^, Georg M. Guebitz^1,2^

*^1^Austrian Centre of Industrial Biotechnology (ACIB), Tulln, Austria*

*^2^ Institute of Environmental Biotechnology, University of Natural Resources and Life Sciences Vienna, Tulln, Austria*

*^3^ Universita degli Studi di Napoli Federico II, Dipartimento di Scienze Chimiche, Napoli, Italia*

*^4^ Institute of Biogeochemistry and Pollutant Dynamics, ETH Zurich, Zurich, Switzerland*

*^a^ present address: Richard Bittner AG, Feldkirchen, Austria*

*^b^ present address: Glanzstoff Industries GmbH, St.Poelten, Austria*

*^*^* ***Correspondence:***

*Alessandro Pellis Ph.D.*

*alessandro.pellis@boku.ac.at*

# Supplementary Figures

| 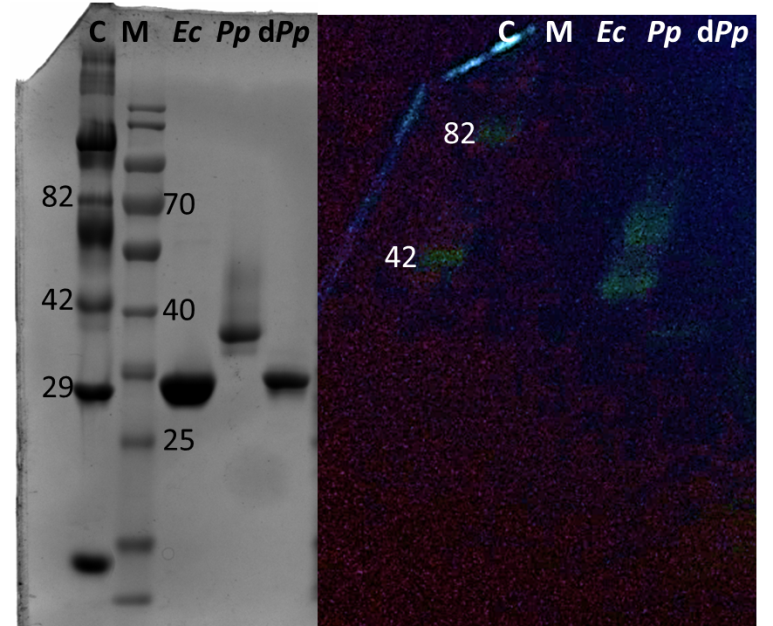 |
| --- |
| Figure S1. Glycostain gel analysis of *E. coli* and *P. pastoris* expressed Thc_Cut1. Left= SDS PAGE gel, Right= Glycostain gel; same samples were applied on both gels; C= Candy cane protein marker; M= peqGold protein marker IV; Ec= *E. coli* expressed Thc_Cut1; Pp= *P. pastoris* expressed Thc_Cut1; dPp= *P. pastoris* expressed Thc_Cut1 deglysolyated with Endo Hf. |

| 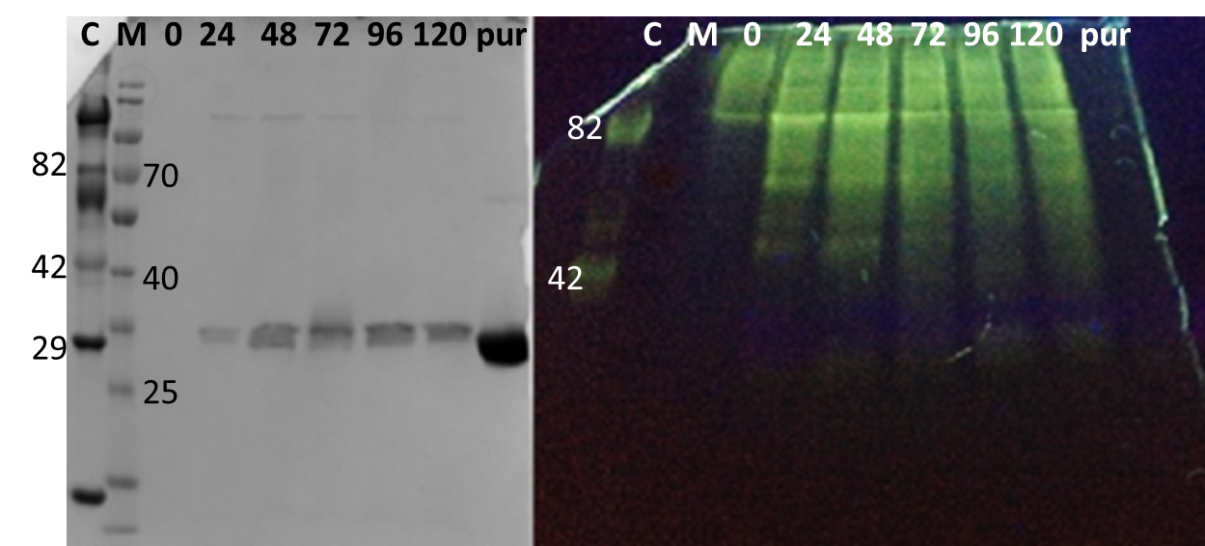 | |
| --- | --- |
| Figure S2. SDS-PAGE (12%) (left) and Glycostain gel (right) of *P. pastoris* fermentation supernatant samples withdrawn at different time points after induction during expression of Thc_Cut1_koST. C= Candy cane protein marker, M= peqGold protein marker IV, #= time after induction in h, pur= ÄKTA purified enzyme. | |
| 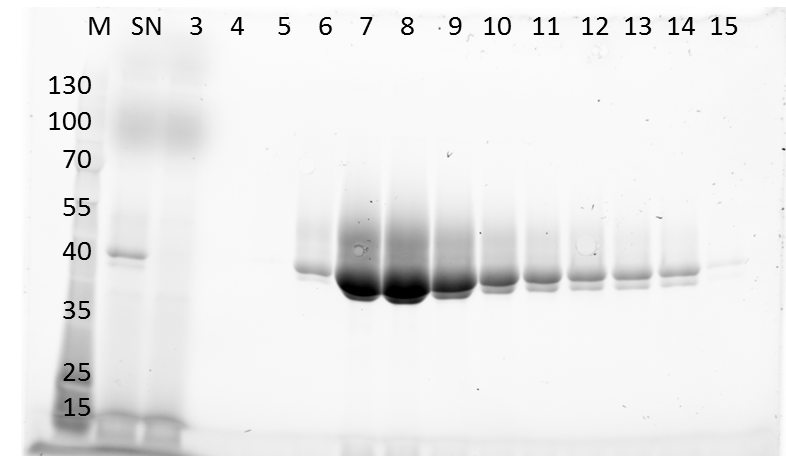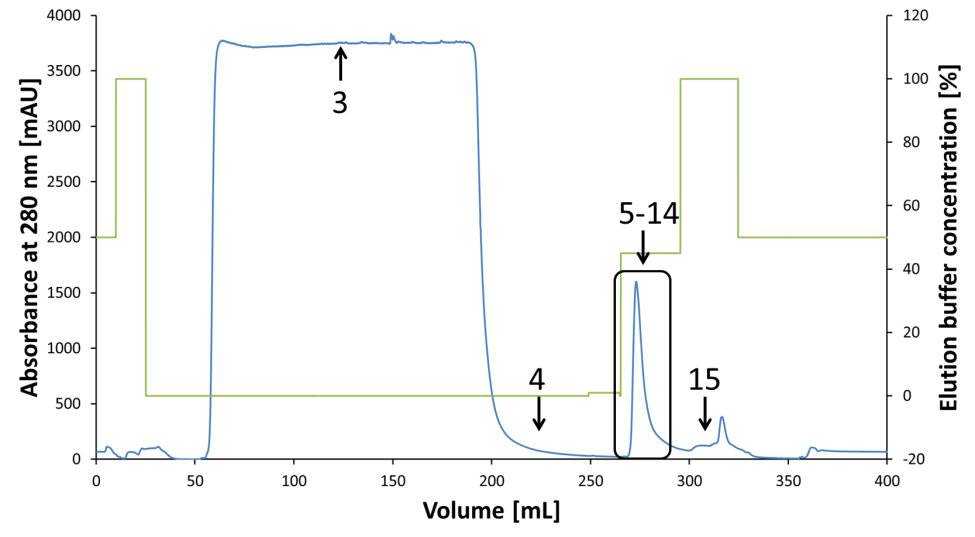 | |
|  | |
| Figure S3. Purification of Thc_Cut1. Top: IMAC chromatogram, blue= absorbance at 280nm [mAU], green= elution buffer concentration [%]; Bottom: SDS-PAGE (12%) of fractions during purification; M= peqGold protein marker IV, SN= supernatant before purification, #= fractions according to chromatogram above. | |
|  | |
| 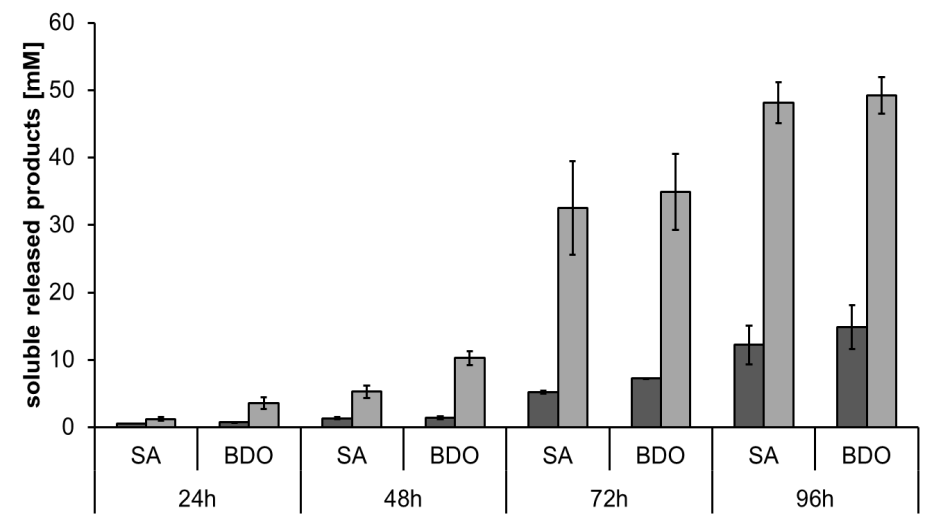 |  |
| **Figure S4.** Concentrations of soluble released products succinic acid (SA) and 1,4-butanediol (BDO) upon enzymatic hydrolysis of PBS films by Thc_Cut1 (dark grey bars) and Thc_Cut1_ko_ST (middle grey bars). Time scan for 24, 48, 72 or 96 h was performed at 65 °C with 5 µM enzyme in 1 M KPi pH 8.0 with 0.5 x 1.0 cm PBS films at 100 rpm |  |

| 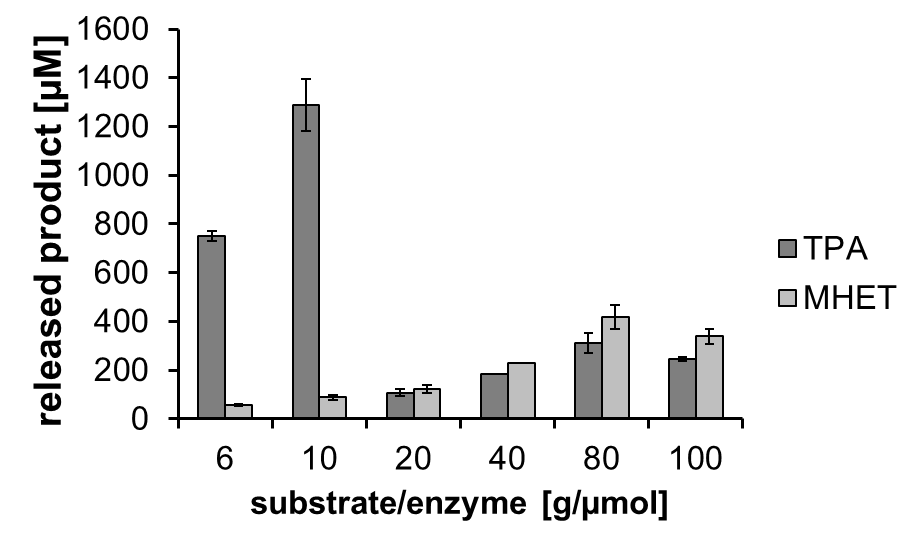 |
| --- |
| 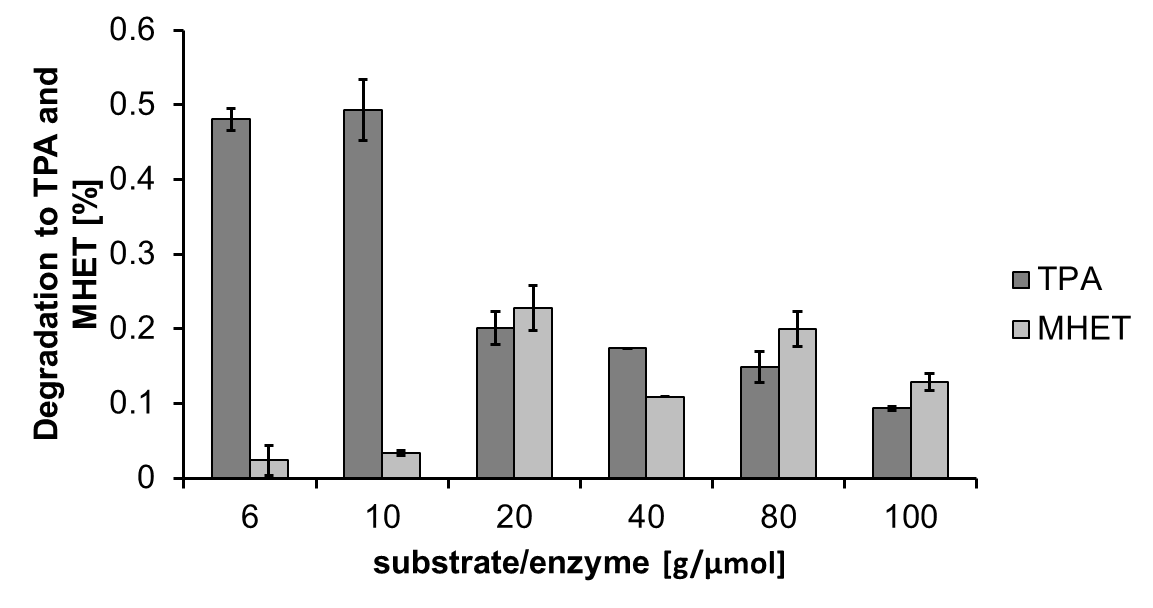 |
| **Figure S5.** Concentrations (top) and percentage of degradation (bottom) to soluble released products terephthalic acid (TPA) (dark grey bars) and mono(2-hydroxyethyl) terephthalate (MHET) (light grey bars) upon enzymatic hydrolysis of PET powder by Thc_Cut1. Hydrolysis was performed for 24 h at 60 °C in 100 mM KPi pH 7.0 using different substrate-enzyme ratios at 300 rpm. |
